# Successful Intravascular Ultrasound-Guided Transradial Coronary Intervention with a 4Fr Guiding Catheter

**DOI:** 10.1155/2016/6369812

**Published:** 2016-09-08

**Authors:** Yasuhiro Nakano, Kenji Sadamatsu

**Affiliations:** ^1^Department of Cardiovascular Medicine, Saga-Ken Medical Centre KOSEIKAN, Saga, Japan; ^2^Department of Cardiovascular Medicine, St. Mary's Hospital, Kurume, Japan

## Abstract

Minimizing the catheter size can reduce vascular access complications and contrast dye usage in coronary angiography. The small diameter of the 4Fr guiding catheter has limited the use of several angioplasty devices such as intravascular ultrasound (IVUS) in the past. However, the combination of a novel IVUS catheter and a 0.010 guidewire makes it possible to perform IVUS-guided percutaneous coronary intervention (PCI) with a 4Fr guiding catheter. We herein report the case of a 51-year-old man with silent myocardial ischemia who underwent IVUS-guided transradial PCI with a 4Fr guiding catheter.

## 1. Introduction

Minimizing the catheter size can reduce vascular access complications and contrast dye usage in coronary angiography (CAG) [1]. Intravascular ultrasound- (IVUS-) guided percutaneous coronary intervention (PCI) is associated with significantly lower rates of adverse clinical events compared with angiography-guided PCI [2]. Although the small diameter of a 4Fr guiding catheter has several critical limitations in the use of angioplasty devices such as IVUS, the combination of a novel IVUS catheter and a 0.010 guidewire may make it possible to perform IVUS-guided PCI with a 4Fr guiding catheter.

## 2. Case Presentation

**Figure 1:**
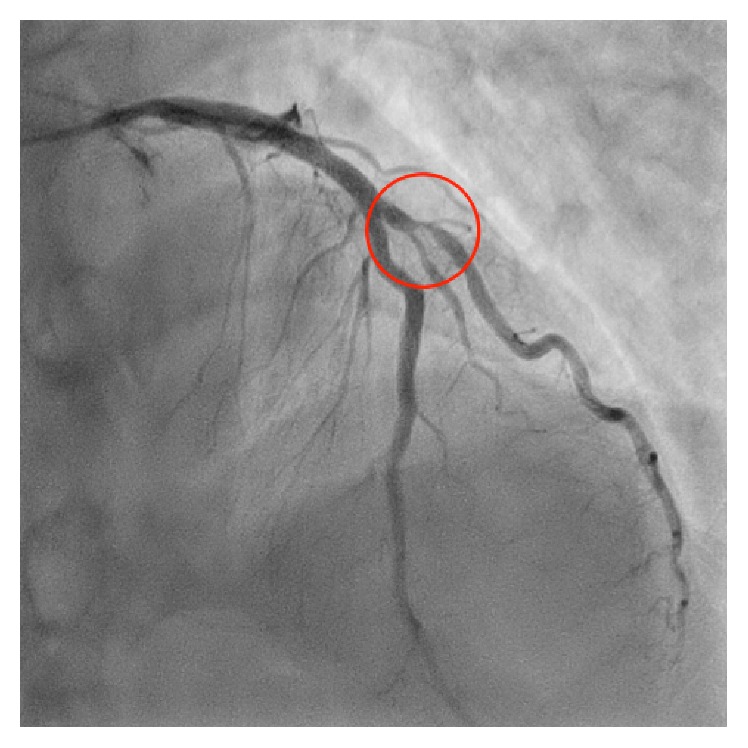
Anterior-posterior cranial view showed the significant stenosis of the proximal portion of the second diagonal branch before percutaneous coronary intervention.

**Figure 2:**
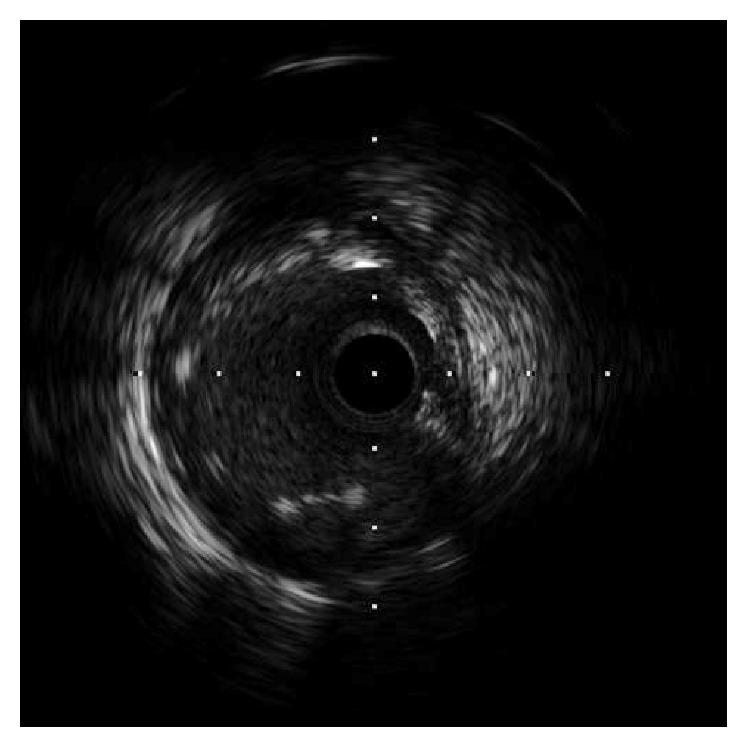
Intravascular ultrasound imaging revealed incomplete apposition of the stent struts in the proximal edge.

**Figure 3:**
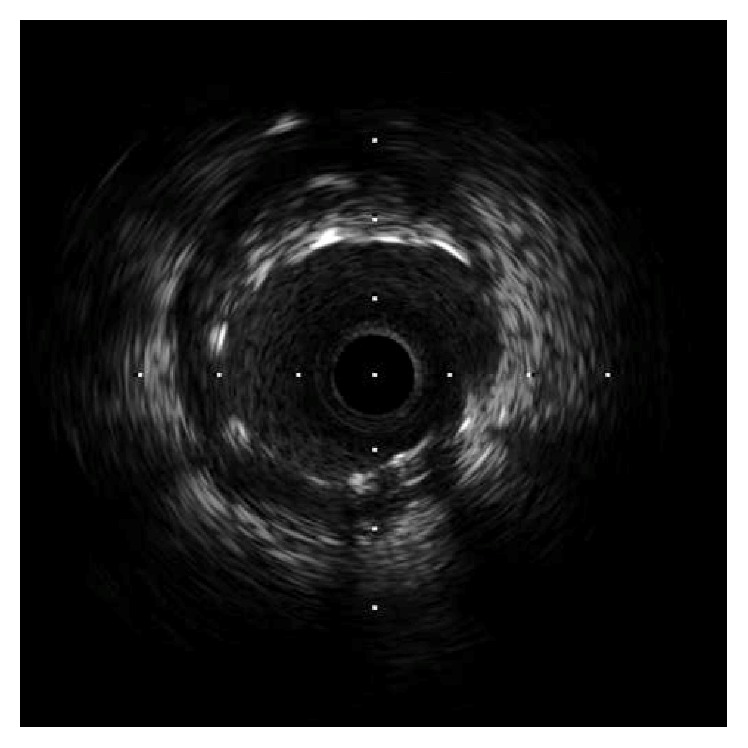
Repeat intravascular ultrasound imaging revealed improved apposition of the stent strut.

**Figure 4:**
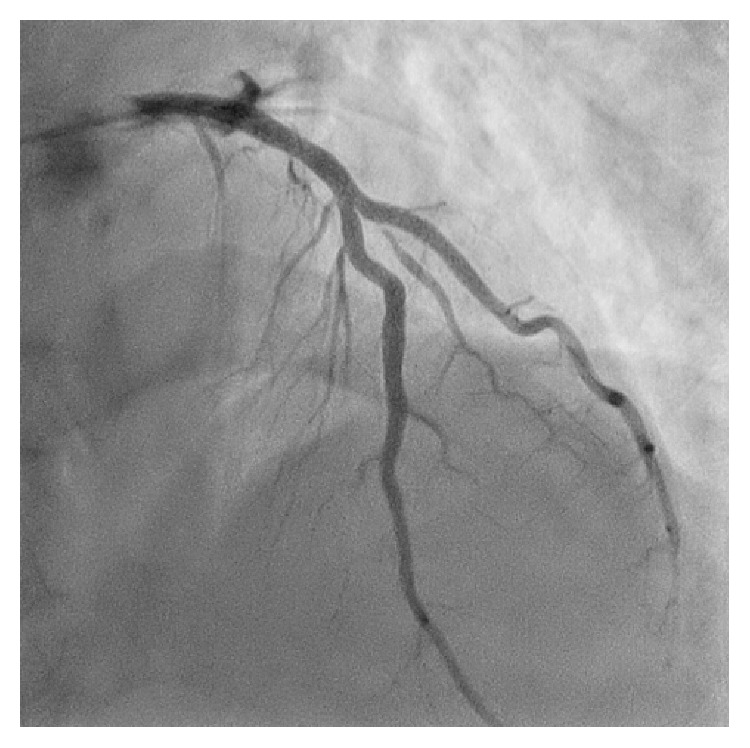
Anterior-posterior cranial view showed the widely dilated proximal portion of the second diagonal branch after percutaneous coronary intervention.

A 51-year-old man was admitted to our hospital for acute inferior ST-elevation myocardial infarction 1 month previously, and emergent coronary angiography revealed in-stent restenosis in the distal right coronary artery and severe stenosis of the proximal portion of the second diagonal branch (D2). In-stent restenosis was the culprit lesion and was treated with a drug-coated balloon. One month later, the patient was readmitted to our hospital for elective PCI to the D2 lesion (Figure 1). A 4Fr BL3.5 guiding catheter (KIWAMI Heartrail, Terumo, Tokyo, Japan) was used to engage the left coronary artery and a 0.010 inch guidewire (Decillion HS, Asahi Intecc, Aichi, Japan) was advanced across stenosis into the distal D2. An IVUS catheter (OptiCross®, Boston Scientific, Natick, MA) was passed smoothly in the 4Fr guiding catheter and the D2 lesion. IVUS images demonstrated that the stenotic lesion was an eccentric fibrous plaque with superficial calcium. After predilation of the lesion with a 2.5 × 15 mm scoring balloon (Scoreflex, OrbusNeich, Hong Kong, China), a 3.0 × 16 mm everolimus-eluting stent (Promus Premier, Boston Scientific, Natick, MA) was deployed successfully in the D2 lesion. IVUS images revealed incomplete apposition of the stent struts in the proximal edge (Figure 2); therefore, post-dilation of the stent proximal edge was performed using a 3.5 × 8 mm noncompliant balloon (Powered Lacrosse 2, Goodman, Aichi, Japan) at a maximum of 16 atm. The final IVUS findings revealed that the apposition of the stent strut was improved (Figure 3), and final angiography showed good results (Figure 4).

## 3. Discussion 

TRI can reduce vascular access complications and contrast dye usage in coronary angiography [3]. However, radial artery diameters can vary widely from 1.5 to 4 mm [4], and a large-sized catheter for patients with small radial arteries may cause radial artery occlusion [5]. Thus, the small profile of the catheter, especially <6Fr catheters, referred to as slender PCI, has a favorable impact on vascular access complications [6].

**Figure 5:**
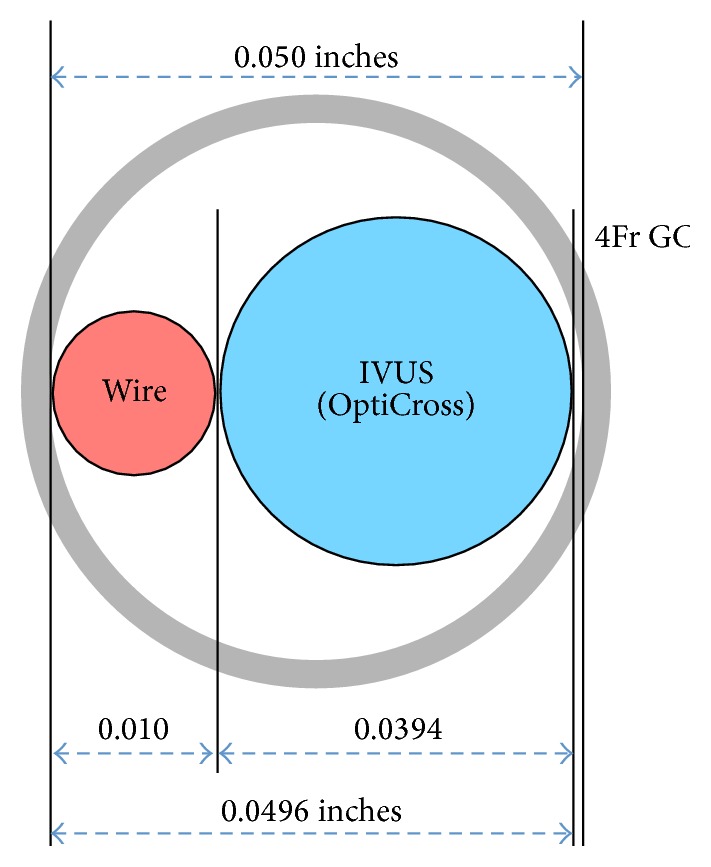
A schematic overview showed the respective diameter of 4Fr guiding catheter, 0.010 guidewire, and intravascular ultrasound catheter.

A 4Fr guiding catheter has been developed to facilitate TRI [7]. However, a 4Fr guiding catheter does not allow successful passage of an IVUS catheter because the inner diameter of 4Fr guiding catheters is limited to 0.050 inches. Recently, a novel IVUS catheter (OptiCross) with a thinner profile has become available. The shaft diameter of the IVUS catheter is 0.0394 inches, and the exit port diameter is 0.0413 inches; thus, the IVUS catheter enables us to use a 4Fr guiding catheter in combination with a 0.010 guidewire (Figure 5). Slender TRI with the 4Fr guiding catheter is less invasive, and moreover, IVUS guidance can be achieved safely and reliably for patients with coronary artery disease. Additionally, we can apply this method to the 4-in-5 mother-child technique for treatment of complex coronary lesions [8]. Slender PCI with a 5Fr guiding catheter is becoming widespread in use; however, one of the critical disadvantages of 5Fr guiding catheters is insufficient backup; thus, it is difficult to use the technique for some complex lesions. The 4Fr double-coaxial technique (mother-child technique) is one of the solutions to overcome the problem of backup, and the method in this report further supports the use of IVUS in addition to the 4-in-5 mother-child technique for slender PCI in the treatment of complex coronary lesions.

There are several limitations associated with this method. First, the only currently available IVUS catheter for this method is OptiCross due to the limitation of the catheter diameter. Other IVUS catheters are too large to pass through the inner lumen of the 4Fr guiding catheter. Second, a 0.010 guidewire has decreased torque and support; however, the contemporary types of this wire have similar operability to conventional 0.014 guidewires. Third, this method does not allow the IVUS marking technique, which is a way to obtain the optimal angiographic view for stent deployment and appropriate making of the exact position of interest by using IVUS transducer. At present, this method is limited to the selected case. Fourth, the novel introducer sheath of the 5Fr Glidesheath Slender (Terumo, Tokyo, Japan) may be an alternative method to 4Fr PCI. The sheath has the same inner lumen size as that of a conventional 5Fr sheath, combined with an outer diameter similar to that of a conventional 4Fr sheath [9]. However, the Glidesheath Slender is not available in some countries and more costly compared with conventional introducer sheaths.

## 4. Conclusion

The combination of a novel IVUS catheter and a 0.010 guidewire makes it possible to perform IVUS-guided PCI with a 4Fr guiding catheter. This method enables less invasive and safer TRI for patients with coronary artery disease.
